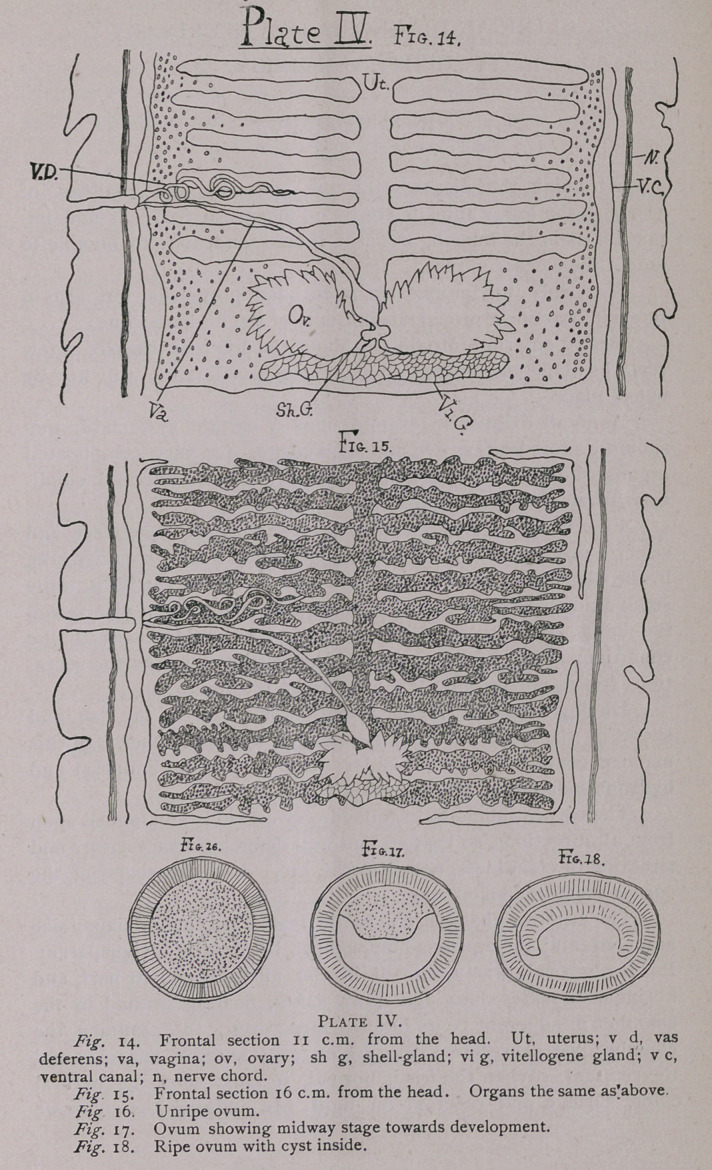# On the Anatomy of Tænia Crassicollis Rud†“G. Brown Goode” Prize Thesis, Wesleyan University, 1893.

**Published:** 1894-02

**Authors:** A. E. Loveland

**Affiliations:** Assistant in Biology, Wesleyan University, Middletown, Conn.


### THE JOURNAL
OF
### COMPARATIVE MEDICINE AND VETERINARY ARCHIVES.

VOL. XV. FEBRUARY, 1894. No. 2.

### † ON THE ANATOMY OF TÆNIA CRASSICOLLIS RUD.

BY A. E. LOVELAND, B.A.,
Assistant in Biology, Wesleyan University, Middletown, Conn.

SYNONYMY—A. ADULT STAGE.*

1767 *Tænia cucurbitina* Pallas, pro parte.
1782 *T. collo brevissimo* Bloch.
1782 *T. serrata* Gœze.
1782 *T. globulata* Gœze.
1786 *T. moniliformis* Batsch.
1786 *T. serrata* Batsch.
1788 *T. serrata a, cati domestici* Schrank.
1790 *T. serrata b, felis* Gmelin.
1793 *T. felis* Carlisle.
1800 *Alyselminthus serratus* Zeder.
1803 *Halysis serrata* Zeder.
1810 *T. crassicollis* Rudolphi.

B. LARVAL STAGE.‡

17— *Vermis vesicularis muris* Hartmann.
1781 *Tænia hydatigena* Pallas.
1782 *Vermis vesicularis tæniaeformis* Bloch.

---

† "G. Brown Goode" Prize Thesis, Wesleyan University, 1893.

* In making up the synonymy of this species, I have accepted as synonyms several names which Rudolphi mentions, although I have not been able to find the articles in which the names were originally used, and so can give them only on Rudolphi's authority.

‡ The above synonymy I accept from Rudolphi, as I have been able to find very few of the books in which these names are given.

1782 *T. vesicularis fasciolata* Gœze.
1788 *Hydatigena tæniaeformis* Batsch.
1788 *Vesicaria tæniaeformis* Schrank.
1788 *Vesicaria muris* Schrank.
1790 *T. vespertilionis* Gmelin.
1790 *T. hydatigena* Gmelin.
1790 *T. murina* Gmelin.
1795 *Hydatula macrocephala hepatis musculi* Viborg.
1803 *Cysticercus tæniæformis* Zeder.
1803 *C. vespertilionis* Zeder.
1810 *C. fasciolaris* Rudolphi.

HOSTS—A. ADULT WORM.

*Felis catus.*—Wild cat (of Europe).
*Felis domestica.*—Domestic cat.
*Felis concolor.*—Puma, Cougar, Panther.
*Felis macroura.*—'Kuichua.'
*Felis maniculata.*—Egyptian cat.
*Felis melivora.*
*Felis onca.*—Jaguar.
*Felis mitis.*
*Felis tigrina.*—Margay (?).
*Putorius erminea.*—Stoat, Ermine.

B. LARVAL STAGE.

*Mus decumanus.*—Brown or Norway Rat.
*Mus musculus.*—House Mouse.
*Mus rattus.*—Black Rat.
*Arvicola amphibius.*—Water Vole.
*Arvicola arvalis.*—Field Vole.
*Plecotus auritus.*—Long-eared Bat.

GEOGRAPHICAL DISTRIBUTION.

Europe.—Germany, found by Leuckart; Austria, found by Rudolphi; Italy, Grassi and Parona; France, Ralliet; England, Hassall; Denmark and Iceland, authority of Raum.

North America.—United States.*—Pennsylvania, at Philadelphia, by Leidy; Washington, D. C., Stiles, Curtice, Hassall; Connecticut, at Middletown, by Loveland.

Asia.—Persia, by Baird.

* Although very few printed records are to be found of the presence of this parasite in this country, the species is evidently quite common, as a number of college zoologists have mentioned the form to me.—C. W. S.

* BIBLIOGRAPHY.

(1684) REDI, FRANCESCO.—Osservazione di Francesco Redi intorno agli animali viventi, che si trovano negli animali viventi in Firenze (quoted from Rudolphi).

(1729) REDI, FRANCESCO.—Opisculorum pars tertia, sive animalculis vivis quæ in corporibus animalium vivorum reperiuntur observationes. Lugd. Bat. (Rudolphi).

(1767) PALLAS, PETER SIM.—Neue Nordische Beyträge. Bd. I., p. 47; Tab. 2; fig. 1, 2. A. B. (Rudolphi).

1782 BLOCH, MARCUS ELIESER.—Abhandlung von der Erzeugung der Eingeweidewürmer, p. 19, n. 18, Tab. 6, fig. 1–3. (Rudolphi).

1782 GOEZE, JOHANN AUGUST EPHRAIM.—Versuch einer Naturgeschichte der Eingeweidewürmer thierischer Körper, p. 337, Tab. 24. fig. 1–6; Tab. 25 A, fig. 1–5. (Rudolphi).

(1786) BATSCH, AUGUST JOHANN GEORGE CARL.—Naturgeschichte der Bandwurmgattung ueberhaupt und ihrer Arten insbesondere, nach den neuern Beobachtungen in einem systematischen Auszuge, verfasst von Aug. Joh. George Carl Batsch. Mit 5. Kupfert, Halle. 298 P. 8 p. 135, n. 9, fig. 59. *T. moniliformis.* P. 138, fig. 19, 32, 61, 63, 67. *T. serrata.* (Rudolphi).

(1788) SCHRANK, FRANZ VON PAULA.—Verzeichniss der bisher hinlänglich bekannten Eingeweidewürmer, p. 36, n. 107. (Rudolphi).

(1790) GMELIN, JOSEPH FRID.—Systema Naturæ, p. 3067, n. 31, p. 3068, n. 32. (Rudolphi).

(1793) CARLISLE, ANTHONY.—Transact. Societatis Linneæ. II t. 25, fig. 11. *T. felis.* London. (Rudolphi).

(1800) ZEDER, JOHANN GEORGE HEINRICH.—Erster Nachtrag zur Naturgeschichte der Eingeweidewürmer von J. A. E. Goeze, mit Zusätzen und Anmerkungen, herausgegeben von J. G. H. Zeder. Mit 6 Kupfert. Leipzig, p. 286. (Rudolphi).

(1803) ZEDER, J. G. H. Anleitung zur Naturgeschichte der Eingeweidewürmer, Bamberg. P. 363, n. 50. (Rudolphi).

1810 RUDOLPHI, CARL ASMUND.—Entozoorum sive Vermium Intestinalium Historia Naturalis. Vol. II, p. 173.

1845 DUJARDIN, FELIX.—Histoire Naturelle des Helminthes, p. 603.

1850 DIESING, CARL MORITZ.—Systema Helminthum.

*I have been unable to obtain those works the dates of which are enclosed in parentheses.

1855 LEUCKART, R.—Erziehung des *Cysticercus fasciolaris* aus den Eiern von *Tænia crassicollis* (Leipzig); Zeitschrift für Wissenschaftliche. Zool. VI.

1857 KÜCHENMEISTER, FRIEDERICH.—Parasites of the Human Body. Translated by Lankester.

1873 NITSCHE, HEINRICH.—Zeitschrift für Wissenschaftliche. Zool. Vol. XXIII, Heft. 2.

(1874) SCHIEFFERDECKER.—Jenaische Zeitsch. f. Naturwissensch. Bd. VIII.

1879 GRASSI ET PARONA.—Sopra la *Tænia crassicollis;* Atti soc. Ital. sc. natur. XXII. 1 taf. Milano, p. 207–219.

1880 MONIEZ, R.—Essai monographique sur les Cysticerque ; Travaux de L'Institut Zoologique de Lille, p. 60.

1882 PERRONCITO, EDOARDO.—I Parassiti Dell 'Uomo E Degli Animali Utili, p. 208.

1882 ZÜRN, F. A.—Die thierischen Parasiten auf und in dem Körper unserer Haussäugetiere.

1883 RAUM, J.—Beiträge zur Entwickelungsgeschichte des *Cysticercus fasciolaris.* Dissertation, not published in any journal.

1886 LUDWIG, H.—Leunis' Synopsis der Thierkunde. Vol. II, p. 867.

1886 RAILLIET, A.—Éléments de Zoologie Médicale et Agricole. Paris. P. 235, 236, fig. 117. *Cysticercus fasciolaris.*

1888 VOGEL, LEONARD.—Ueber Bau und Entwickelung des *Cysticercus fasciolaris* Rud. Inaug.-Diss. Erlangen. Osterwieck Harz, Buchdruckerei von A. W. Zickfeldt.

1892 NEUMANN, L. G.—Treatise on the Parasites and Parasitic Diseases of the Domesticated Animals. P. 467–469, fig. 244. (Translated from the French by Fleming).

1892 BLOCHMANN, F.—Ueber Sommer's sog. "Plasmatische Längsgefässe" bei *Tænia saginata* Goeze, und *Tænia solium* L.; Centralblatt für Bakteriologie und Parasitenkunde. No. 11-12, p. 373–379.

1893 STILES, CHARLES W.—Bemerkungen ueber Parasiten—17: Ueber die topographische Anatomie des Gefässsystems in der Familie Tæniadæ; Centralblatt für Bacteriologie und Parasitenkunde. No. 14-15, p. 462, 463.

### HISTORICAL REVIEW.

Francesco Redi (1684) seems to have been the first who published upon this species. Rudolphi refers to a mention of this worm in the "Observations upon Parasites of Living Animals" by Francesco Redi in 1684.

Peter Sim. Pallas next described it in 1767 in the "Neue Nordische Beyträge," and catalogued it "sub *Tænia cucurbitina.*"

M. E. Bloch (1782), in his "Abhandlung von der Erzeugung der Eingeweidewürmer," names this species "*Tænia collo brevissimo.*"

In brief, he describes it as follows: The head does not rest on a long thread-like neck, but is sessile upon the broad but very minutely segmented body itself. The head is surrounded with thirty-six hooks in two circular rows, those of the inner row being the largest. The four suckers are plainly visible to the naked eye; the anterior segments are broader than long, while the posterior are longer than broad. The genital openings lie on both sides of the body in alternate segments. It lives in the cat; the longest specimens measuring two feet in length, and from two-twelfths to three-twelfths inches in breadth.

J. A. E. Goeze (1782) describes the worm in general like Bloch, specifying five brief designations:

1st. The gradual running of the segments directly into the head.

2d. At the posterior end the irregular genital openings can be seen with the naked eye.

3d. The genital openings are not alternate in some cases, but successive.

4th. The posterior segments are long and narrow.

5th. The head is unusually large, with four suckers and a double crown of hooks.

He names it "*Tænia serrata,*" because of the serrate appearance of the ends of the segments, each segment extending and overlapping the next one, and ending on both sides in very sharp points, the whole effect being much like a carpenter's saw.

Rudolphi also mentions in his synonymy of this worm Goeze's description of *T. globulata*, but this latter is evidently another species, if I understand Goeze's description.

A. J. S. C. Batsch (1786) calls this worm "*Tænia moniliformis,*" although he refers to it as "*Tænia serrata*" of Goeze. In this case, as well as in the case of the following six authors, I simply quote Rudolphi, as Dr. C. W. Stiles, who kindly procured for me most of the remaining works, was unable to get these for me.*

---

*I want to express my deep gratitude also to Dr. Stiles, not only for placing his laboratory, together with his resources for obtaining the necessary literature, entirely at my disposal, but also for his assistance all through the investigation, and especially for his aid in adding to and correcting the article before publication.

Franz von Paula Schrank (1788) mentions the species in his catalogue of the worms known up to this year (1788).

J. F. Gmelin (1790) also speaks of it in his "Systema Naturæ" as "*Tænia serrata*," and again as "*Tænia moniliformis*."

Anthony Carlisle (1793) called it "*Tænia felis*," in his publication in the "Transactions of the Linnean Society."

J. G. H. Zeder (1800) named it "*Alyselminthus serratus*" in his "Erster Nachtrag zur Naturgeschichte der Eingeweidewürmer von Goeze." Again, in 1803, Zeder, in his "Anleitung," describes it sub "*Halysis serrata*."

Carolo Asmundo Rudolphi (1810), in his classical work upon intestinal parasites, gives the above bibliography of the species, and, because of the specially broad neck, names it "*crassicollis*" (broad neck). His description is as follows:

Characteristics: "Tænia capite crassiusculo cum collo brevissimo continuo; rostello cylindrico; articulis anticis transversis, insequentibus cuneiformibus, postice acutis, reliquis oblongis; foraminibus marginalibus vage alternis."

In further details his description is not different from Bloch and Goeze given above, except in these particulars: he denies that the genital openings may be sometimes successive on the side, but says they are always alternate; each margin that has the genital openings is broader than the opposite; head, rostellum and hooks larger than in most tape worms; neck continuous from the head, and of nearly same breadth as body, which shows the discrimination of this from other worms. Thus Rudolphi names it *crassicollis* because it was broader necked than most tape worms, and from his time the name *crassicollis* has been maintained, although *Taenia serrata*, so named by Goeze, should by priority have the precedence.

M. Felix Dujardin (1845), in his "Histoire Naturelle des Helminthes," gives the length as 300 to 400 millimetres; he believes the species is identical with *T. laticollis*.

C. M. Diesing (1850) describes *T. crassicollis* in his classification of Helminthology; measurements: length one foot and over; breadth, middle segments one-third of an inch, last segments one-sixth of an inch.

Habitation, cat family; found in different species at different times of the year; *Felis concolor*, April, May and November; *Felis melivora*, March and April; *Felis onca*, November; *Felis paradalis*, April; *Felis macroura*, April; *Felis tigrina*, June, September and October.

Up to this time nothing was known concerning the larval stage of the worm, as the great truth later discovered by Frederich Küchenmeister had not yet dawned upon the scientific world. and it was the similarity between the *Cystice-cus fasciolaris* and the *T. crassicollis* that first led Küchenmeister to the conception that *Tæniæ* were but adult forms of *Cysticerci* which in their larval stages lived in the bodies of animals upon which the hosts of the adult *Tæniæ* preyed. In this way *Cysticercus fasciolaris* encysted in the liver of a mouse could easily be transmitted to the intestine of a cat, and there the worm become the adult *crassicollis*. Besides other experiments Küchenmeister performed that of feeding mice with the eggs of *T. crassicollis*, and afterwards reversing the feeding, proved conclusively that these *Tæniæ* were but adult forms of *Cysticerci*.

About this same time (1853) R. Leuckart was experimenting with parasites, and to apply Küchenmeister's theory made a trial of feeding mice with the eggs of *Tænia crassicollis ;* and in a letter to von Siebold (1855) he says he successfully infected four out of five white mice with the eggs of the *T. crassicollis*, and upon opening the mice after four months found them in the cyst stage in the liver, and in less developed stage in the omentum.

Frederich Küchenmeister (1857), in describing the cyst form, says the segmented joints are developed somewhat, but the vesicle which is large in other cyst forms becomes dropsically degenerate in this cyst. He was never able to discover the six embryonic hooks on the oncosphere.

Heinrich Nitsche (1873) is the first who studied the muscular mechanism of the rostellum and neck. He says : the rostellum is the crown of the head and bears the hooks ; it is an elastic cushion and cannot contract of itself, but does so only by means of the sheath of muscles surrounding it. This rostellum can be pushed out and in by the muscles, but it is fastened in such a way that the contraction of the trunk has no influence at all upon the position of the hooks, for these can be turned in and out as well when the trunk is withdrawn as when it is extended. The movement of the hooks is caused by the drawing in of the rostellum.

Radial muscles, longitudinal, and circular muscles : the circular muscles and the longitudinal muscles below the rostellum being contracted, at once draw down the rostellum, changing its quiescent form to a meniscus form, which is open towards the front, so that the hooks on the border assume an almost vertical position. The anterior surface of the rostellum being covered only by skin is more movable than the posterior and therefore the rostellum is pulled in and down.

But besides the longitudinal muscles the radial muscles act on the anterior surface, and move the hooks downwards in proportion as the diameter of the posterior end narrows by being drawn inwards, thus having the effect of straightening the hooks. The radial muscles bend in a bow-shaped curve away from the periphery to the centre, and then out again to the periphery on the other side, crossing one another in their course through the series of layers.

Schiefferdecker (1874). (I was unable to find any matter written by this author).

Grassi et Parona (1879) give the length of the worm from 25 to 60 cm., rostellum having from 40 to 52 hooks, the longer ones 0.3 mm. to 0.32 mm. long, the shorter ones 0.18 mm. long, the rostellum itself 0.39 mm. in diameter. Grassi found the smallest mature *T. crassicollis* yet mentioned, namely, 15 cm. long. It was found in the stomach of *Felis catus* freshly killed, about two cm. from the pyloric end, firmly adhering, and still alive.

It is interesting that Grassi and Parona thus give record of finding the *T. crassicollis* in the stomach. Neumann and Perroncito also state the same. Its usual habitation, according to Grassi and Parona, is the middle part of the small intestine. It thus occurred in 90 out of 100 post-mortem examinations. Grassi also found reported in a medical journal a case of *T. crassicollis* in the stomach. Groves states that tape-worms are not uncommon in the stomach of horses; Diesing also cites that the *Tænia plicata* (=*Anoplocephala plicata*) is often found in the stomach of the horse, that the *T. pectinata* has been found in the stomach of the *Arctomys marmota*, and that the *T. salmonis* occurs in the stomach of the Olmul. Yet, despite these facts, the lodgement of *T. crassicollis* in the stomach is undoubtedly an exceptional and rare occurrence.

Another strange thing found by Grassi and Parona was that several tape-worms were discovered by them extended through the intestinal wall into the body-cavity, and some were even found lying free in the body-cavity, bearing the appearance of having been dead for some time. But this was undoubtedly due to a perforation of the intestine near the spot where the animals were attached, which let them out into the cavity. These authors give no internal anatomy of the worms, only discussing these two points: First, their living in the stomach; secondly, being found free in the body-cavity.

R. Leuckart (1879-1886), in works published between these periods, describes the rostellum and scolex the same as Nitsche, but disagrees with that author in that the rostellum has powers of contraction itself, and is not merely an elastic cushion. He says that besides the longitudinal and circular muscles, outside the rostellum is a layer of circular muscles directly ensheathing the rostellum, and these were overlooked by Nitsche.

R. Moniez (1880) adds a few facts. The cysts found in the liver of the mice were noticed as follows: those buried within the liver were much pinched and elongated, while those on the surface were expanded and filled with a liquid accumulated within the cyst-wall. The latter were ovoid in shape and from three to four millimetres in diameter. In the interior is often found a very fine granular mass, produced by the degeneration of the substance within (or the excrescence), which can be colored easily for examination by ammoniated picro-carmine. The cyst covering or shell is a modification of the connective tissue of the liver.

Comparing the *Cysticercus fasciolaris* with the *C. pisiformis*, the former does not leave the liver, but remains there, if not disturbed, until the mouse dies, while the latter leaves the liver and fixes itself to the peritoneum, casting off its surrounding cyst. The young *Tænia* is invaginated in the vesicle, and protrudes a very littie, but is diminished by the contraction of the muscles of the worm, which are continuous with those of the vesicle. This is the reason the cyst is so large and the vesicle so small. According to Leuckart the segments of the Cysticerci all change and new segments take their place.

Dr. Edoardo Perroncito (1882) gives the measurements as 150 to 600 mm. in length and 4 to 6 mm. broad anteriorly. Hooks 40 to 52 in number, the larger ones 0.30 to 0.32 mm, the shorter 0.18 mm. long. The diameter of the suckers 0.39 mm. The genital openings are irregularly alternate. Lastly he describes a case occurring in Fuelli, Italy, of a *T. crassicollis* being found in the stomach of a cat.

F. A. Zürn (1882) makes the following points: first, that the hooks are used not only for holding fast to the intestinal wall, but for boring purposes as well; secondly, that the larvæ of these tape-worms are also segmented worms (*Cysticercus fasciolaris*) and live in the liver of mice.

J. Raum (1883) states that Wepfer (1688) was the first who noticed this worm, but did not attempt to identify it or name it. Raum himself made an extended study of the habits and life of *C. fasciolaris*. He made a successful infection of mice with eggs of adult *T. crassicollis*, though by some mistake in the method of inoculation many of the mice died the first week, some of which showed signs of cysticerci, while in others there was no trace of infection at all. Only 5 per cent., however, failed to show signs of the appearance of the cysticerci.

In Copenhagen similar experiments showed only a failure of 5 per cent., while in Iceland 23 per cent. failed

Blumberg gave record of 28 per cent. failing, but he experimented with only fourteen mice.

Raum's study of the growth and development of the cyst in the liver of the mouse is briefly as follows: After five to seven hours no embryos can be seen in the stomach, hence they lose their shells in two, three, or four hours. Only four times did he find the embryo or oncosphere in the large intestine, and each of these times only a few hours after feeding, showing the cyst to be really on its passage to the small intestine, while in the small intestine itself could be seen numbers of them, so many that he counted ten in his microscope field. Out of several hundred intestinal examinations, only twice did he find the unmistakable boring hooks of the oncospheres, and this will not be considered remarkable when it is remembered that Küchenmeister was never able to see the hooks at all at this stage. One important point remains to be noticed from his observations, namely, that he never found any oncospheres in the capillaries of the mesentery, but found three in the portal vein, one at nine, another at twenty-seven, and another at fifty-two hours after infection.

H. Ludwig (1886): "*Tainia*" (a band) is the name of a tapeworm in Aristotle; *tænia*, it is called in Pliny. "*Crassicollis*" from crassus, thick; and collum, neck. Length 10-60 cm.; head, semispherical, 1.5 mm. broad; double row of hooks (48-52), neck 1.8 mm. broad; ripe segments as long as broad, or even longer; edges projecting.

A. Railliet (1886): *T. globulata* Goeze; *T. crassicollis* Rudolphi. Length 15 to 60 cm.; a double crown of hooks 26 to 52 in number (often 34), the large ones 0.38 to 0.42 mm. long, the shorter ones 0.25 to 0.27 mm. long. The posterior segments measure 8 to 10 mm. in length, by 5 to 6 mm. in breadth. Eggs globular, from 0.031 to 0.037 mm. in diameter. Larval form found in the liver of rats, Surmulots (Norway rats), common mice, meadow-mice, water-rats, and even in the horse-shoe-bat, according to Bloch. The cysticercus is remarkable for its elongated form, and the smallness of the development of the caudal vesicle. The head is invaginated, the body simply corrugated, but with no trace of genital organs. *T. semiteres* Baird, he thinks, is but a monstrous form of the *T. crassicollis*.

As the subject of his thesis for a doctor's degree Vogel (1888) prepared numerous investigations upon the *Cysticercus fasciolaris*, and he gives a very exhaustive discussion of the subject. From this I have merely chosen the most salient and important points.

First some eggs of *T. crassicollis* were fed to mice, and to determine whether they lost their shells in the stomach or not, other eggs were placed in gastric juices artificially prepared in the laboratory at a temperature of 35 degrees C., and left there from one hour to two days, but in no case, when examined under a microscope, had they completely lost their shells. Vogel concluded from this that the shells were not completely lost till they had passed through the large intestine, though they were probably weakened and partly lost in the stomach. As to the passive wandering of the embryos through the blood claimed by Küchenmeister and Heubner, and denied by Raum, Vogel saw nothing of the kind, though after twenty-eight days he saw a cyst lying in the wall of a blood-vessel. In no case either did he see any signs of hooks, from which this stage is called the oncosphere, although Raum, and Raum alone, has noted such an observation. Upon examination of the liver after four or five days, the round bodies of the cysts were plainly visible; after six to eight days no differentiation was found in the egg-cyst, though the tough outer coat could be noticed; after nineteen days the first appearance of the muscles, after twenty-eight days the cysticercus is plainly seen within, after seventy-four days the head begins to be seen and the segments to show, and after eighty-four days the invaginated head is well shown. Goeze has recorded as high as fifty specimens of the cyst form in the liver of a mouse, and of the adult worm the author (Vogel) found from three to fifteen specimens in one cat. Seventy-one mice were fed with the eggs of *T. crassicollis*, and in no case after the cysts were fully developed did he find more than two in each liver, though in the first stages they were numerous. The first few days after the feeding the liver became covered with numberless minute white spots, which in fourteen or fifteen days grew more heterogeneous and larger. A liver of sixteen days showed the cysts as large as the head of a small pin, and were scattered all about the peritoneal covering of the liver. By the twenty-fifth day they had grown to the size of a large pin-head, and in fifty to seventy days had reached the size of two large pin-heads. By this time the number had rapidly diminished, the strongest driving out the weakest, though the mice had not shown a sign of disease during the whole time, nor did they for the five months the experiments were in progress.

In the laboratory of the Zoological Institute of Berlin are two specimens of *Cysticerci*, the one 45 cm. long, the other 34 cm. long, and the segments in both are very strongly developed.

The body of *Cysticercus fasciolaris*, according to Vogel, has a hyaline exterior membrane, then three layers of cuticle,* the first light colored and thick, the second light but thinner, and the third still lighter and thinner. Very minute canals or pores enter the interior. Next to the cuticle-layer is a very thin layer of circular muscles, and beneath these a sub-cuticle layer of cells which lie with their points towards the outside in a longitudinal direction. Below this a thin layer of muscular tissue interspersed with calcareous bodies, and under this a stout layer of thicker muscles which radiate towards the ends and move the whole body.

The excretory canals of the *Cysticercus*, like those of the *Tænia*, are four in number. They empty near the bladder into two main tunnels, and these two are joined immediately into one central median canal which opens into the bladder by a single excretory pore. The outer ventral canals he describes as connected by a "*Ringgefäss*" or circular canal running at the posterior border of each segment, but he figures this connective canal as a single straight-coursed canal, hence it is evident that Vogel did not clearly understand this part of the anatomy, because in the first place his description does not agree with his figure, and in the second place both figure and description are incorrect. The inner and smaller dorsal canals run parallel with the larger outer ones, but have no transverse canals joining them together; on the contrary, they run completely through the worm from anterior to posterior end without a break. Both canals give off fine capillaries which pass into the parenchyma, and anastomose in all directions. In the head they unite just below the rostellum in many folded serpentine coils, and end blindly just below the suckers, between them and the rostellum.

Nervous system: two main trunks that connect at the head, passing like a bow over the top of the rostellum † In the head it

*I could find but two of these, though there may be three.

†Vogel here must have meant the polster, for the bow unites just below the rostellum, but just over the top of the polster.

is a trifle larger than in the trunks, but nowhere is there any swelling that approaches a ganglion. Both nerve-trunks pass down behind the ventral canals, and there is but one single trunk without branches. They end bluntly in the tissue of the last segment. Nitsche and Steudener saw this trunk divided into two parts by muscles passing between the fibers, Steudener even seeing three divisions, and Vogel saw the same number as Steudener.

L. G. Neumann (1892): Worm 15 to 60 cm. long; head 1.7 mm. broad, with a powerful rostellum; a double crown of 29 to 52 hooks (most frequently 34), the largest .38-.42 mm. long, the smallest .25-.27 mm. long. Posterior segments 8-10 mm. long and 5-6 mm. broad. Ova globular 0.031 to 0.037 mm. in diameter. Cystform is remarkable for its elongated form and smallness of bladder. Length of whole cyst when stretched out 3 to 20 cm.

Diseased cat shows symptoms as follows: Gradual diminution in size, abdomen retracts, complete loss of appetite, slight diarrhoea at the commencement, then constipation, abundant salivation, sometimes spasmodic contraction of the muscles of the upper lip, great prostration and loss of sight; hearing somewhat affected; vomiting which gives temporary relief.

Nervous phenomena: epileptiform convulsions and a violent gastric catarrh.

F. Blochmann (1892), in the "Centralblatt für Bacteriologie und Parasitenkunde," mentions the peculiarity at the junction between the transverse canal and the lateral canals. It is this: that out of each chief (ventral) canal there springs towards the median line two branches which unite together after a short distance, and pass through transversely as one canal. Thus a section of the transverse canal made close to the lateral canals, shows two cross sections of the transverse branches, while a section still nearer the median line shows only one section of the transverse canal. Between these two branches runs the dorsal canal, so that in a transverse section of the worm through one of the proglottides, the dorsal canal lies in a triangle, the lateral sides of which are formed by the roots of the transverse canal, and the base by the ventral canal.* (Fig. 12 shows this).

Charles W. Stiles (1893), in the "Centralblatt," confirms the observations of Blochmann as to the division of the transverse canals into two roots where they join the ventral canal, and adds

* Vogel it seems had already noticed a peculiarity in the transverse canals at either end, but he describes the form as circular, and as we have seen, did not understand the exact conditions at all.

further that both of the genital canals cross from the median field to the margin on the ventral side of the nerve and longitudinal canals

Specific Name.—In justice to Gœze the name "*Taenia serrata*" should be retained, for he named it thus in 1782, and Rudolphi named it *crassicollis* in 1810, but as I am not monographing the genus *Tænia*, I will retain the name now commonly accepted, i. e., Rudolphi's "*Tænia crassicollis*."

It was named by both Pallas and Bloch prior to 1783, but "*Tænia cucurbitina*" was already used for a species found in another animal, and the name of Bloch, "*T. collo brevissimo*," is trinomial, and hence cannot be accepted.

ORIGINAL OBSERVATIONS.

From my own observations on this species I have obtained the following results :

There were eight of the worms living in the intestine of the cat when I found them. These eight measured from 17 cm. to 31 cm. long; average 23½ cm. Some that I had access to in the laboratory of the Bureau of Animal Industry, at Washington, D C. belonging to Leidy's collection, measured 12-15 cm. in length. The width varies from 2.5 mm. to 5 mm., the number of segments 160-220; the first segments 1 to 2 mm. broad, and the posterior segments 3-5 mm. broad, and 6-10 mm. long.

The external appearance is as in Fig. 1, Plate 1, the special points of interest being : the thick neck, the first segments wide but extremely short, these gradually growing longer, until at the posterior end they are longer than broad, the serrate appearance of the segments which led Gœze to name the worm *T. serrata*, this being caused by the pointed ends of each segment overlapping the succeeding one. Each segment has a genital opening on the side, just anterior to the centre, but in no case did I find the openings perfectly regular in alternation as has been described before, but as often successive as they were alternate, sometimes as many as six occurring in succession (more frequently two or three) on the same side. The last segment has two free openings to the exterior from the excretory system.

The head or scolex is about the same width as the neck, from 1 to 1.7 mm. broad, with a powerful rostellum bearing a double row of hooks, in the specimens I examined either 17 or 18 in each row, the larger ones measuring 0.32 to 0.42 mm. in length, and the smaller ones 0.18 to 0.28 mm. in length, bearing also four suckers at each corner, measuring 0.30 to 0.50 mm. in diameter.

I will mention here that I found also among Leidy's specimens at Washington in three samples of the liver of rats 18, 20, 26 specimens of *Cysticercus fasciolaris* respectively.

SCOLEX AND HOOKS.

The scolex or head is represented by Fig. 5. The hooks are situated upon the very crown of the head, fastened firmly in the rostellum. Each hook has a posterior root that is embedded in the rim of muscles surrounding the rostellum, and also an anterior root which is nearly as long as the pointed portion called the prong, this anterior root passing directly int › the muscular tissue of the rostellum itself, and is there attached to the muscles (Figs. 2 and 3). Figs. 3 and 4 show sections through the rostellum at the very base of the roots of the hooks. The rostellum itself is more or less free upon the main supporting sheath of muscles, resting upon a cushion of elastic muscles called the polster, the rostellum being more strictly the portion in front of the suckers. This latter can be pulled in and down, or pushed out at will, the polster readily acting with it; thus the hooks resting horizontally (Fig. 5) are acted on in the manner of a lever, and when the rostellum is pulled in, the anterior root is pulled down and the projecting prong raised, and when the rostellum is pushed out the prong is lowered. By this we see how the animal holds on to the wall of the intestine. The muscles of the rostellum radiate from the centre outwards towards the periphery as described by Nitsche, and as he further described, it does not move itself, but only moves as the sheath of muscles outside and below contract or expand. The rostellum does, however, have the power of contracting its muscles centrally, to cause a slight compression. This rostellum itself is practically an elastic cushion of muscle in which the hooks work, and is in turn supported below by the polster, another ball of muscles which are radial and transverse, and consist of five layers as shown in Fig. 8. Nitsche has described the action of these.

The suckers, four in number, situated at four angles make the head appear quadrangular in shape, and are composed of powerful muscles. There are two principal layers, as shown in Fig. 7.

The neck is but an elongation of the body in structure and appearance; the first segments are very minute in size, and of the total 200 or so segments, the first 100 lie within the first 4 or 5 cm. behind the scolex.

EXCRETORY SYSTEM.

The excretory system consists of four lateral canals, two on each side, which run continuously through the body from anterior to posterior. The inner or dorsal canals are completely continuous, but the outer or ventral canals are connected at the division line between each segment by transverse canals as shown in Fig. 9, which is an ideal section drawn to show the relation of these canals with one another and with the nervous system. The four canals, dorsal and ventral, finally empty into two main trunks in the last segment and through these open to the exterior. At the anterior end is a large ring-canal (Figs. 6 and 9), which completely surrounds the polster and lies just at the base of the hooks and the rostellum. This is connected with the ventral canals on either side, and probably with the dorsal canals also, though I was unable to trace the latter to the ring-canal and be sure they were connected with it. The ventral canals, as Vogel describes, are apt to pursue a more or less serpentine or winding course just before they are joined to the ring-canal because of the shortened and contracted condition of the segments in the neck.

Blochmann and Stiles have both shown the peculiarity in the connection between the transverse and ventral canals, *i. e.*, the triangular formation produced by the dividing of the transverse canal at either end into two roots, and through the space between the roots and the ventral canal the dorsal canal passing; and I noted the same thing (Fig. 12). Both these systems give off fine capillaries that pass into the tissue and anastomose in all directions. In regard to Vogel's statement that some authors had claimed these capillaries end in ciliated cells which receive the excretions, I could see no trace of cilia, though they may nevertheless be present, but I believe that the cell tissue of the body absorbs the food and passes the excretions to the excretory system. The structure of the organs themselves is nothing but a tough, structureless cuticular membrane, which has no cell tissue at all.

### NERVOUS SYSTEM.

The nervous system is very simple and consists of two nerve trunks running outside of and parallel to the excretory ducts (Fig. 9). Towards the head these swell out into somewhat larger trunks, and unite just below the rostellum in the shape of a bow. This uniting point is the nearest approach to a ganglion that there is, and from this knot branches go out to the suckers, and up to the muscles of the rostellum.

At the posterior extremity the trunks end bluntly in the tissue of the last segment. I could nowhere distinguish any nerve-cells or anything that looked like a nerve-cell in the whole nervous tissue, it simply being a barren bundle of fibres. Nowhere did I see the trunk divided by muscles or organs, as mentioned by Vogel, but ever one continuous trunk.

### MUSCLES.

The muscular system of the head, including the rostellum, is the most complex, and that has been described above.

In the body proper there are two principal systems of muscles; transverse and longitudinal. Both are situated in such a manner that they enclose the genital organs in a kind of sheath in the form of a flattened cylinder, whose cross-section is an ellipse, as shown in Fig. 11. The transverse muscles are shown in the figure in their positions, and their dorso-ventral radiations at the ends into the outside tissue. The longitudinal muscles run just exterior to the transverse, and form bands about these both of frontal and sagittal muscles, though the latter have to pass directly through the radiating transverse muscles to some extent (Fig. 11). The frontal muscles are in four, sometimes five, separate divisional bands formed one within another about the flat sides of the cylinder (Fig. 10), but the sagittal or dorso-ventral sheath is more of a compact band composed, however, of numerous smaller stripes. This latter sheath encloses and completely surrounds the nerve trunk at either end, and so lies just exterior to the excretory ducts, the latter being shown by Fig. 11 to lie just within the cylindrical sheath. Fig. 10 is taken from a longitudinal section showing the four layers of frontal muscles, and also the structure of the body wall without.

Besides this principal system of muscles is an outer system consisting of minute bands of muscles that run both longitudinally and transversely, following the cuticle layers, and just within them, completely around the whole body, shown also in Figs. 10 and 11.

### GENITAL ORGANS.

The genital system is best described by a series of figures, in order to show the gradual development. First a segment five centimeters from the head shows as follows :

Fig. 13. The ovary consists here of two lateral parts and is very large, the uterus on the other hand quite small and having only one branch which is sufficient to contain the ova at this stage. The vitellogene gland is prominent, but the shell-gland is not shown, it being hidden by the ovary.

Fig. 14. Segment 11 cm. from the head. Here we note the increasing branches of the uterus, the gradual absorption by it of the segment, the ovary becoming crowded and apparently smaller. The shell-gland is shown just below the ovary.

Fig. 15. Here the segment is 16 cm. from the head and very nearly mature. The uterus has become many branched and occupies almost the whole segment. I found these branches generally numbered 12 in the matured segments, though occasionally there appeared to be 13, on each side.

The eggs crowd the uterus of the ripe segments in countless numbers and are 0.03 mm. to 0.04 mm. in diameter. They are globular in form, and the shell or coating seems to be composed of a very thin, delicate outer shell and a more or less radiate and very thick inner shell. This inner portion appears to be of a tough membraneous structure. In perfectly ripe ova the hemispherical formed embryo of the worm may be plainly seen lying within the egg. Fig. 18 shows one of the ova very highly magnified with the cyst within. Figs. 16 and 17 show earlier stages respectively in the development.

The testes are always congregated towards the sides of the segment, and become rarer towards the centre; they are on the dorsal side of the segment always, while the female organs are on the ventral side. Figs. 13 and 14 show this.

The cell tissues have been more or less shown in the preceding figures. The body-cells are transparent, have a nucleus about half the size of the cell, and of darker body than the cell. These are scattered all through the tissue, both central and peripheral portions. Besides these are the pointed cells of Vogel, which lie just within the cuticular layers, completely surrounding the whole animal, and point always to the exterior. Fig. 10 shows these and also the different cuticular layers pointed out by Vogel and which are the same in the adult.

Very conspicuous throughout the muscular portion of the worm are the calcareous bodies which are oval in shape and appear much like cells. Towards the anterior portion of the body, and especially near the head, these bodies are very regular in grouping, a transverse section showing two principal chains, in rows one exterior to the other, following around the body between the genital cavity and the body wall. In the posterior segments they are of much greater number, are situated indiscriminately throughout the muscular tissue, though congregating perhaps in greater degree towards the exterior, are of larger size, and show a more concentric structure, as if they had grown by concentric additions to an inner kernel.

### SUPPLEMENTARY NOTE.

By C. W. Stiles.

While reading the proof of the above article at Mr. Loveland's request, I noticed that an interesting point in connection with the muscles of the neck and head has apparently escaped his attention, and so far as I recall at the present moment, has not been recorded by any author. I will therefore take the liberty of adding a short note covering the point in question, and based upon observations which I made about four years ago, but have not as yet published.

Starting with Loveland's description of the musculature of the body as noticed in the segments, we shall see, by following the various systems into the head, that a complete change in their arrangement takes place upon reaching the neck. The longitudinal muscles split up into four bundles, one dorsal and one ventral bundle of equal size, and two smaller lateral bundles of equal size; these four bundles or groups extend into the head, running between the suckers and gradually diminishing in size as the fibres insert. Hand in hand with this grouping of the longitudinal fibres, the sagittal fibres become more highly developed and the lateral groups of longitudinal fibres are each bounded medially by a layer of these sagittal fibres. A corresponding change takes place in the transverse system of muscles, the ventral fibres gradually turning ventrally at the sides of the worm, and the dorsal fibres turning dorsally. In this way we find eight systems of muscles in a transverse section just below the suckers, *i. e.* (1) a dorsal longitudinal bundle, bounded by (2) a dorsal transverse system which is convex towards the ventral surface ; (3) a corresponding ventral longitudinal bundle, bounded by (4) a ventral transverse system convex dorsally ; (5) a dextral longitudinal system bounded medially by (6) a dextral sagittal system convex sinistrad ; (7) a corresponding sinistral longitudinal bundle bounded medially by a sinistral sagittal system convex dextrad.

In connection with the presence of *T. crassicollis* in the stomach, I might add that Hassall recently examined a cat in the Bureau Zoological Laboratory, and found a specimen of this species in the stomach. Two days previously the cat, which showed signs of gastritis, had vomited a *T. crassicollis*, and upon his being brought to us dead, Hassall found five more specimens of this worm present.

*Fibre zibethicus* (The musk rat) may be added as a new host for the larval stage (recently found by Hassall).

## Figures and Tables

**Plate I. f1:**
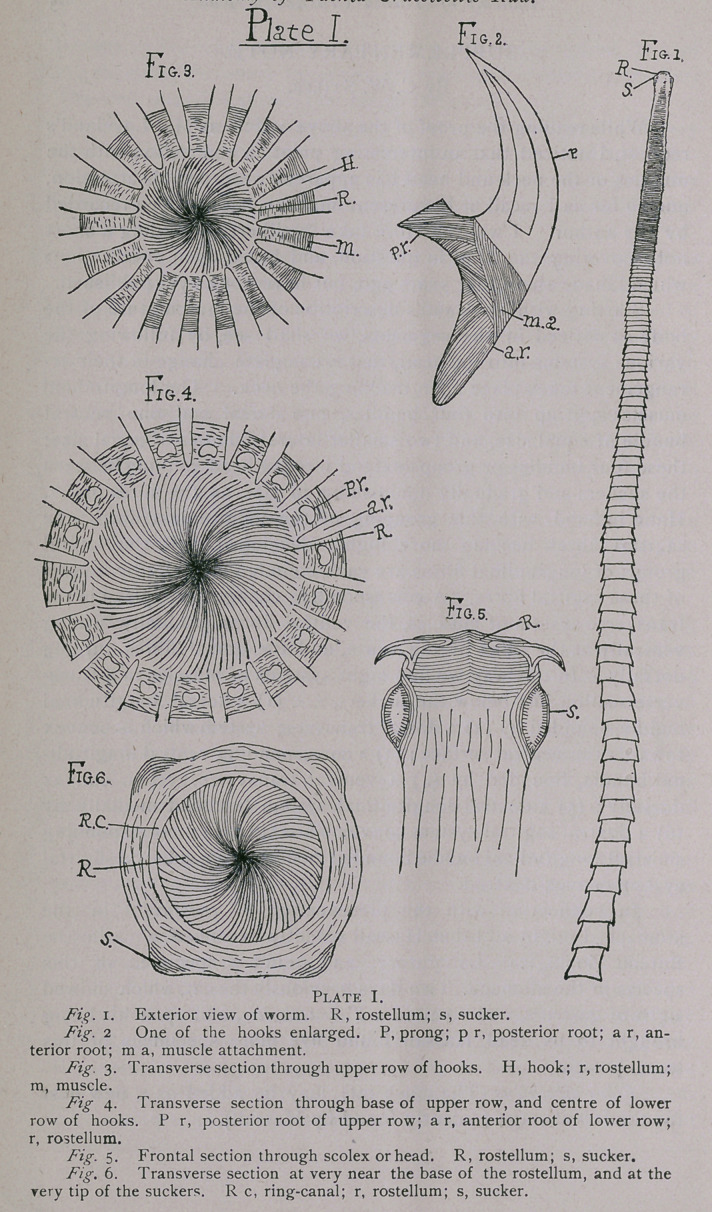


**Plate II. f2:**
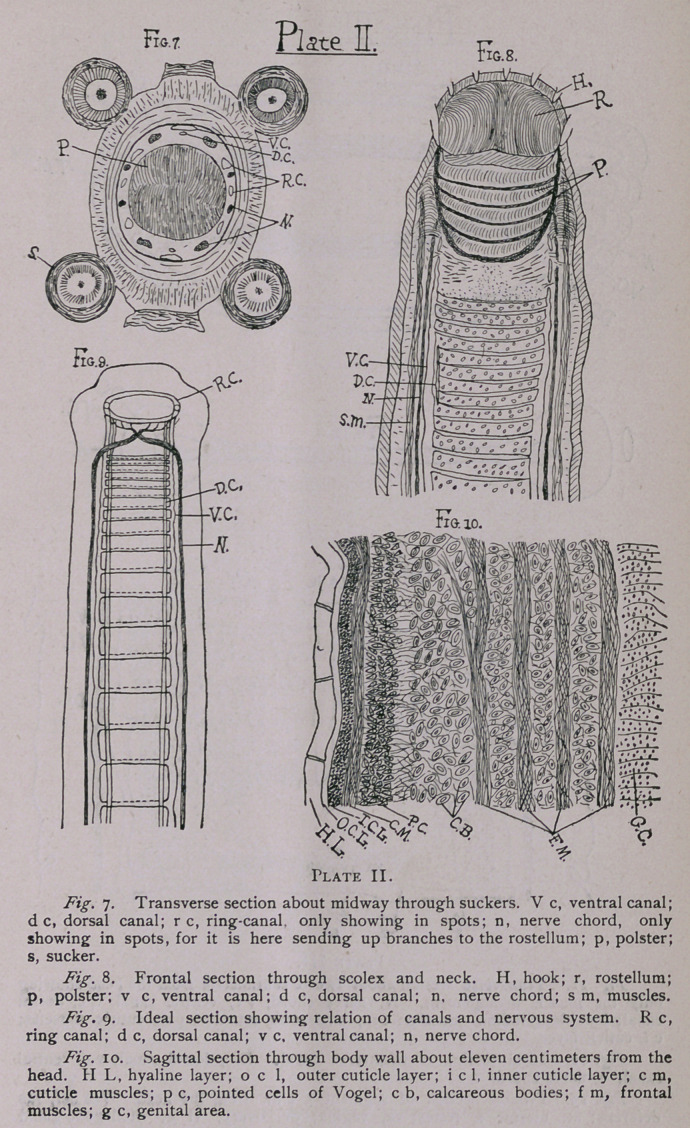


**Plate III. f3:**
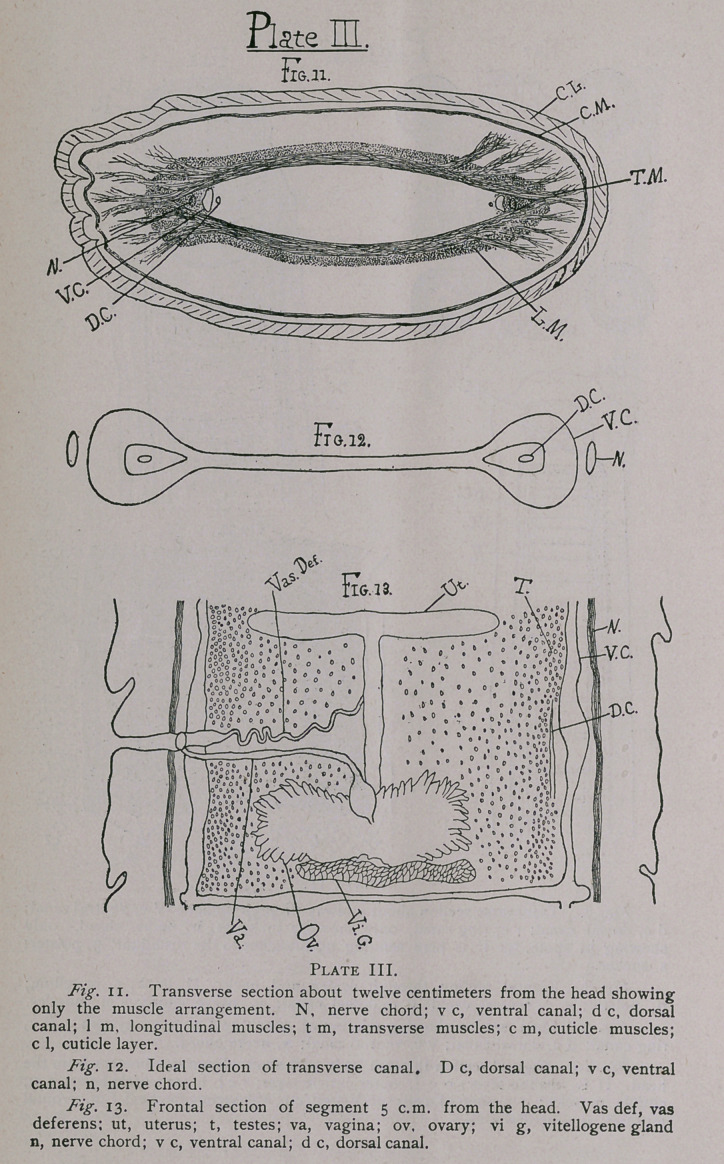


**Plate IV. f4:**